# To the Editors of the Medical and Physical Journal

**Published:** 1804-06-01

**Authors:** E. Hardman

**Affiliations:** Manchester


### *To the Editors of the Medical and Phyſical Journal.*

GENTLEMEN,

THE propriety of the practice of bringing on premature labour at the seventh month, in such cases of distortion of the pelvis, as had, or might require the use of the crotchet, is now so well established, that perhaps an apology may be deemed necessary for again obtruding the subject on your reader's attention; but as the matter is in itself important, and the case I have to offer is conclusive, I shall close my communications regarding it, with the following narrative.

Mrs. M. about twenty-eight years of age, had been twice delivered by the crotchet, at the full time, by a gentleman much employed in midwifery practice, owing to a contraction of the pelvis, which hindered the birth of a full grown fœtus. In her third pregnancy she applied to me, from hearing of my success in similar instances, by bringing on premature delivery. I advised her to submit herself to my directions, and to let me know about the end of the seventh month. She obeyed my injunctions, and I used the proper means for hastening delivery, ordering her at the same time an anodyne mixture to take occasionally, and in which she believed the efficacy of my method to consist. On the fifth day from the rupturing of the membranes the child was born alive, and is now nearly three weeks old, improving daily in strength. The labour was lingering for about forty hours, but not at all strong till within a quarter of an hour before the birth, otherwise nothing remarkable occurred, and she thought herself better the day after than she had formerly done at the end of a week.

Here the child's life was certainly preserved, as well as the sufferings of the mother reduced to nothing comparatively, not to mention her entire safety, an object that may very justly claim the first consideration in every case.

I am, &c.

E. HARDMAN.

*Manchester, May* 3, 1804.

